# Identity by descent and local ancestry mapping of HCV spontaneous clearance in populations of diverse ancestries

**DOI:** 10.1186/s12864-024-11076-6

**Published:** 2025-07-12

**Authors:** Zixuan Yu, Salma Abdel-Azim, Priya Duggal, Candelaria Vergara

**Affiliations:** Johns Hopkins University, Bloomberg School of Public Health, Baltimore, MD USA

**Keywords:** IBD mapping, HCV clearance, Rare variants, GWAS, Unrelated individuals

## Abstract

**Background:**

Acute infection with hepatitis C virus (HCV) affects millions of individuals worldwide. Host genetics plays a role in spontaneous clearance of the acute infection which occurs in approximately 30% of the individuals. Common variants in *GPR158,* genes in the interferon lambda (*IFNL)* cluster, and the Major Histocompatibility complex (MHC) region have been associated with HCV clearance in populations of diverse ancestry. Fine mapping of those regions has identified some key variants and amino acids as potential causal variants but the role of rare variants in those regions and in the genome, in general, has not been explored. We aimed to detect haplotypes containing rare variants related to HCV clearance using identity-by-descent (IBD) haplotype sharing between unrelated cases-case pairs and case-controls pairs in 1,739 individuals of European ancestry and 1,869 African Americans. Additionally, we aimed to detect ancestry-specific effects in African Americans using local ancestry mapping.

**Results:**

We detected 2,370,341 and 1,567,748 individual pairs of IBD segments in the individuals of European ancestry and African Americans, respectively. Individuals of European descent had more segments of longer length compared to African Americans. We did not detect any significant IBD signals in the known associated or new gene regions. We also failed to detect any significant genome-wide local ancestry signals in the African Americans.

**Conclusions:**

IBD is based on sharing of haplotypes and is most powerful in populations with a shared founder or recent common ancestor. For the complex trait of HCV clearance, we used two outbred, global populations that limited our power to detect IBD associations. Overall, in these population-based samples we failed to detect rare variations associated with HCV clearance in individuals of European ancestry and African Americans, and we didn’t detect local ancestry-specific effects associated with HCV clearance in African Americans with our current sample size.

**Supplementary Information:**

The online version contains supplementary material available at 10.1186/s12864-024-11076-6.

## Background

Hepatitis C virus (HCV) infection affects around 200 million individuals worldwide [1, 2]. Approximately 30–50% of HCV-infected individuals spontaneously clear the virus [3–5] while others will experience persistent HCV infection developing chronic hepatitis C, liver cirrhosis, and HCV-related hepatocellular carcinoma [6]. The proportion of individuals who spontaneously clear HCV varies by ancestry [7] and is influenced by age and comorbidities, especially other viral infections including HIV or HBV co-infections.

Host genetics is a key determinant of both spontaneous clearance of HCV infection as well as anti-viral treatment response [3]. Previous genome-wide association studies (GWAS) have identified common variants with large effect in the region of *IFNL4* and *IFNL3* genes, the MHC region, and G-protein-coupled receptor 158 gene (*GPR158*) in populations of diverse ancestry [8, 9]. Collectively, these variants with high minor allele frequency explain 5–7% of the variance of HCV clearance across ancestries [9, 10]. Fine mapping of the MHC signal suggested amino acids in the HLA-DQβ1 molecule as potential causal variants in the region for both populations [11]. In contrast, fine mapping of the *IFNL* locus by sequencing yielded limited information due to the genomic structure with high repetitive segments [12].

Identity-by-descent (IBD) methods leverage genome wide array data to detect haplotype sharing and identify signals and individuals harboring potential rare disease-causing variants which can be followed via sequencing of the target regions [13]. This approach determines whether two purportedly unrelated pairs of individuals in a dataset share segment at a certain genomic position inherited from a common ancestor. Using “pairwise” statistics, the rate of IBD in case-case pairs is compared to that in case–control pairs [14–16] to detect segments with excess of sharing among cases.

IBD mapping has successfully detected rare variants for non-infectious diseases related complex traits such as diabetes, acne, multiple sclerosis, and schizophrenia [17–20] as well as ultra-rare loss-of-function variation associated with blood-related traits in individuals from the UK Biobank dataset [21]. However, the use of IBD to map variants associated with susceptibility to infectious diseases is scarce and, so is the analysis of the effect of rare variants in HCV clearance in populations of diverse ancestry. We aim to detect the effect of rare variation using IBD mapping in a large dataset of unrelated individuals of European ancestry and African Americans with hepatitis C virus clearance and persistence.

## Results

### Study Sample

We performed IBD mapping using GWAS data for HCV clearance and persistence in 3,608 individuals from two genetically determined ancestry groups which are participants of the Extended HCV Genetic consortium, as previously described [8, 9, 22]. This includes 1,869 admixed African Americans (340 with HCV clearance and 1,529 with HCV persistence) and 1,739 individuals of European ancestry including European Americans (702 with HCV clearance and 1,037 with HCV persistence). No new genotyping was conducted for this study and only previously genotyped data is used. The datasets analyzed during the current study are available in the dbGAP repository with dbGaP Study Accession: phs000248.v1.p1 (https://www.ncbi.nlm.nih.gov/projects/gap/cgi-bin/study.cgi?study_id=phs000248.v1.p1). Distribution of the analyzed individuals by genetically determined ancestry group, sex, and HIV infection status is presented in Table 1. The two main principal components of cases and controls in each one of the groups are presented in Supplementary Figure S1.

**Table 1:** Demographic characteristics of the individuals analyzed by genetically determined ancestry groups

Genetically Determined Ancestry Group	N	HCV infection Persistence:Clearance	( +) HIV infection (n, %)	Female Sex (n, %)
Admixed African Americans	1,869	1,529:340	732 (38.6)	624 (33.3)
European Ancestry	1,739	1,037:702	259 (16.1)	544 (31.2)
Total	3,608	2,566:1,042	981 (28.2)	1,168(32.3)

### Genotyping and IBD mapping

After standard GWAS quality control [8], we used 661,397 autosomal single nucleotide polymorphisms (SNPs) to detect IBD segments in cases and controls. We detected 2,370,341 and 1,567,748 unique segments in individuals of European ancestry and admixed African Americans, respectively.

**Fig. 1:**
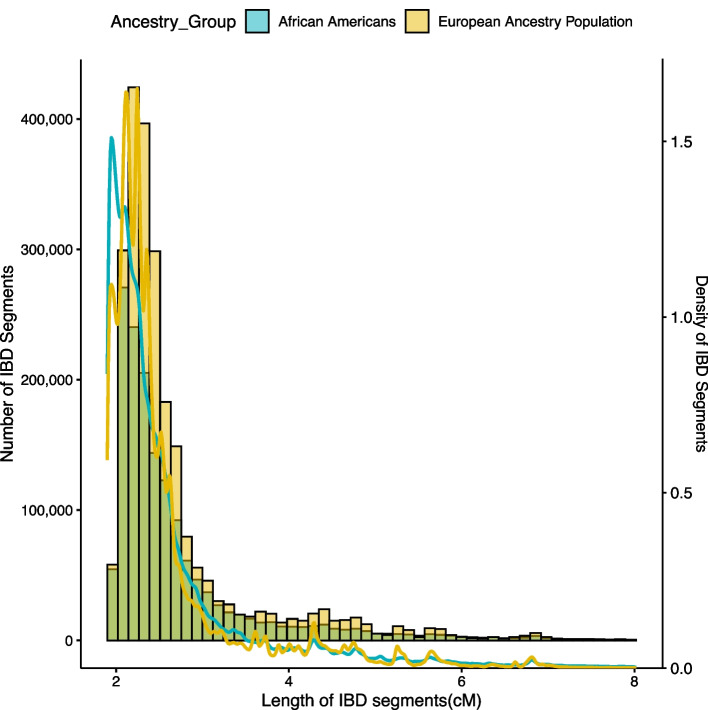
Density plots of the lengths of detected IBD segments in individuals with HCV clearance and persistence from samples of European ancestry and admixed African Americans. IBD segments > 8 cM are not shown

Overall, we found more and longer (on average) IBD segments in the Europeans than in the African Americans (Fig. 1). The majority of the segments were < 5 cM in both populations (99.3% and 97% for individuals of European ancestry and African Americans, respectively) with a mean length of segments of 2.4 cM for both ancestry groups. All detected segments > 2 cM were used in the analysis.

**Fig. 2:**
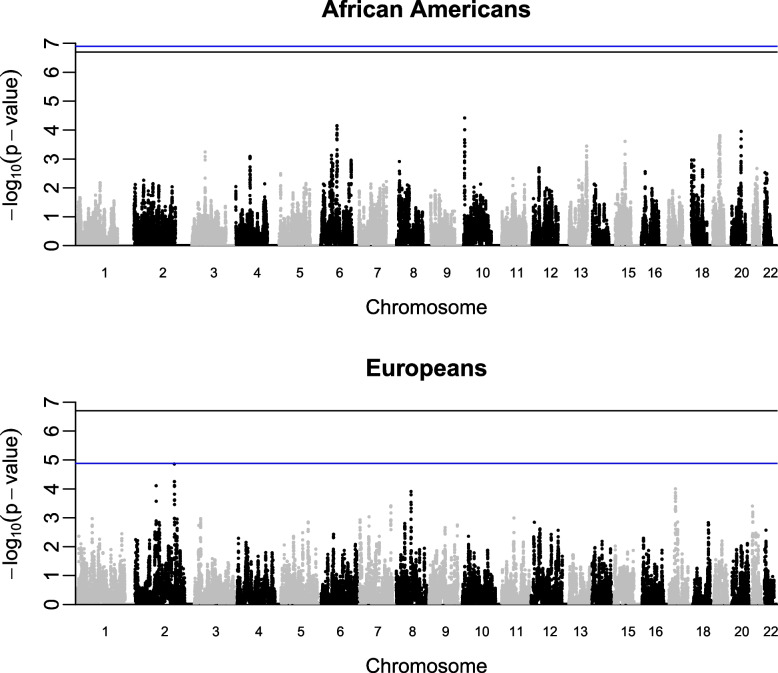
Manhattan plot of the *P* values obtained using IBD permutation analysis in African Americans (Upper Panel) and individuals of European ancestry (Lower Panel). The blue line indicates the genome-wide threshold for each population and the black line is the minimal permutation value obtainable with 5 million permutations (2 × 10^–7^). Each dot corresponds to the *P* value of a genetic marker. Chromosome coordinates are based on GRCh37/hg19 built

For the analysis we used 406,114 and 52,712 case-case pairs of IBD segments in individuals of European ancestry and African Americans, respectively, and 1,148,980 and 469,480 case–control pairs, respectively. Segments > 2 cM were analyzed using a permutation analysis comparing the rate of IBD shared between case-case to case–control to identify IBD alleles in SNPs contained in those segments in each ancestry population. We did not detect any imbalance in the rates of IBD between the case-case and the case–control pairs along the genome (Supplementary Figs. 2 and 3). We observed higher-than-expected IBD rates in the boundaries of the chromosomes and centromeres. This analysis failed to confirm known associated regions in MHC, *IFNL* or *GPR158* loci [8]. We did not detect new regions with GWAS significant associations in either population (Fig. 2).

### Admixture mapping

**Fig. 3:**
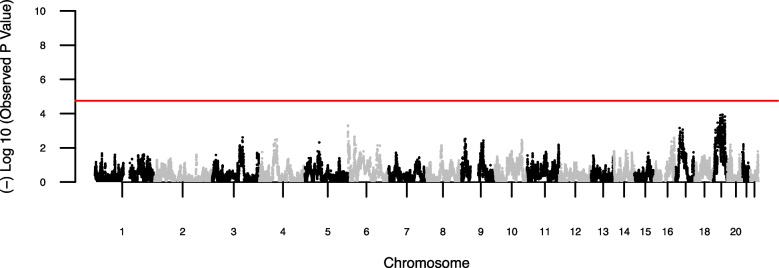
Manhattan plots summarizing the results of the association of local African ancestry and HCV clearance in 1,869 admixed African American individuals (1529 with HCV persistence/340 with HCV clearance). Each point corresponds to the P value for a marker representing a local ancestry window estimated by RFMIX v2. The *P* values are plotted by location of the markers across the genome. The red line represents the level genome-wide significance (*P* value = 1.80 × 10^–5^). No markers exceeded the significance threshold

To detect any differences in local African ancestry between cases and controls and to complement the IBD analysis via admixture mapping, we inferred 25,810 local ancestry segments across the admixed African American genomes. Mean global ancestry proportions for cases and controls were 0.823 and 0.820, respectively. There were no significant differences in the estimates of local ancestry between HCV clearance and persistence in this group (Fig. 3).

## Discussion

Identity by descent mapping did not identify any significant associations for HCV clearance in the European/European-American ancestry or admixed African American ancestry groups. IBD mapping is advantageous at detecting variants where there are multiple rare causal variants clustered within a gene. In this scenario, it is well powered at identifying significant genetic regions [23]. Similarly, IBD mapping may be more powerful for samples from some ancestral populations because of historic bottlenecks leading to more detectable IBD [15]. However, the ability to detect IBD depends on the number of generations to the most recent common ancestor which is reflected in the length of the IBD segments. More recent common ancestry tends to result in longer and more detectable IBD segments [24] which is based on the inferred effective population size of the analyzed population [25]. As expected, in our study, we identified an enrichment of slightly longer segments in the individuals of European ancestry as compared to the African American admixed population. African populations have more haplotype diversity and lower levels of LD compared with non-African populations, such as Europeans [26]. Other factors potentially limiting the power of our IBD analysis include 1) the lower frequency of haplotypes. The frequency of the shared haplotypes is expected to be smaller in outbred populations, and the haplotype is unlikely to be observed twice in independently sampled individuals [16]. 2) Our study also focused on unrelated individuals as appropriate for HCV infection, and the possibility of minimizing errors at phasing is higher in family studies in comparison with unrelated persons and 3) having a denser or increased genomic coverage could improve the ability to detect regions of IBD [15, 16].

Additionally, the simulation study by Browning et al. [23] indicates that the relative performance of IBD mapping and SNP association testing depends on population demographic history and the strength of selection against causal variants. For outbred populations, exceptionally large sample sizes may be required for genome-wide significance unless the causal variants have strong effects [23]. The inability to detect strongly associated regions in this study is likely due to the large diversity of the haplotypes present in these outbred populations especially in the admixed population of African Americans [26, 27]. Moreover, hepatitis C viral infection does not exert a large selective pressure in the host genome to create large conserved and detectable haplotypes shared by cases [28, 29]. Hepatitis C infection is often acquired after childhood through contact with the blood of an infected person via reuse of injecting instruments, unprotected sex, transfusion of unscreened blood and blood products, among others. It doesn’t affect fitness of host alleles given the availability of successful treatment and the chronicity of the condition which allows the survival, reproduction, and mating of the affected individuals. This is in comparison to other infections such as *Plasmodium falciparum* malaria which exerts strong selective pressure on the human genome [30] and results in conserved regions across ancestral populations with a higher homogeneity of haplotypes susceptible to be detected by IBD in unrelated individuals [31]. This difference in pathogen pressure likely limits the utility of IBD for infectious diseases that occur globally, and thus in outbred populations, but without strong selective pressure.

A strength of this study is the consideration of IBD in admixed African Americans using a well-established methodology. However, there are limitations to the success of this approach in admixed population where shared segments could have a European or African origin. Thus, we considered admixture mapping. The evaluation of local ancestry in the admixed African Americans identified no difference in the proportions of African ancestry between cases and controls. Similarly, principal components further support these null results. IBD estimation in admixed populations face additional challenges related with precise modeling of the proportion and components of genetic ancestry which vary between populations, between individuals and through the genome of individual of each admixed population [32]. They depend on the number of parental populations and their effective size, past growth, bottlenecks, founder effects, recent expansion, and number of generations since the admixture event as well as differences in the dynamic of the admixture [15, 16].

## Conclusions

IBD mapping is a complementary method to SNP based association analysis for prioritizing both individual samples and genomic regions harboring rare variants for follow-up with sequencing analysis. Length of the detected segments in this study was slightly different between European ancestry and admixed African American individuals. IBD mapping and local ancestry evaluation for HCV clearance did not detect any associations. Additional approaches are needed to evaluate populations of admixed ancestry. The increase of sample size may be favorable for the detection of associated variants with weak effect, especially in populations with shorter and more diverse IBD segments with low pathogen selective pressure.

## Methods

### Study population

In this study, we analyzed 3,608 individuals from two genetically determined ancestry groups in the Extended HCV Genetics Consortium [8, 9, 22], 1,739 individuals of European ancestry including European Americans and 1,869 admixed African American ancestry. This is a multi-site international consortium including multiple studies from Europe and United States with well characterized HCV infection outcomes. Information about HIV infection status was obtained in the included individuals since it is a determinant of HCV clearance. Each individual study obtained consent for genetic testing from their governing Institutional Review Board (IRB) and the Johns Hopkins School of Medicine Institutional Review Board approved the overall analysis [8].

### Genotyping

Genotyping and quality control has been detailed previously [8]. Briefly, samples were genotyped using the Illumina Omni1-Quad BeadChip array (Illumina) and processed using standard genome-wide association study protocols for quality control [8]. In this study, only autosomal SNPs were used for detecting IBD segments and markers in mitochondrial DNA and sex chromosomes were disregarded. Genetic ancestry and population structure for the Extended HCV Genetic consortium was determined by principal component analysis (PCA) using the smartpca program in EIGENSOFT [33] as previously described [8]. Two main principal components indicate overlapping in the clustering of cases and controls in each ancestry group (Supplementary Figure S1).

### Transformation of datasets and IBD mapping

Datasets for each analyzed group were originally formatted as Variant Call Format (VCF) files. We used Beagle version 5.4 [34] to phase the data and hap-IBD for detecting IBD segments [35] in the complete set of cases and controls in each group separately using default settings. Hap-IBD uses a simple seed-and-extend method. The algorithm finds all seed segments, and it extends each seed segment if there is another long-IBS segment for the same pair of haplotypes that is separated from the seed segment by a short non-IBS gap. A segment may be extended multiple times. When it is no longer possible to extend the segment, the segment is written to the output file if its cM length is greater than the length specified in the settings. The genetic and physical distances were based on build GRCh37/hg19 of the human genome [36]. We used an LD based genetic map as described by Brown et al. [35]. The original map was generated using LDhat as described in reference [37]. Linear interpolation is used to estimate the genetic map positions for any marker whose position is not on the genetic map. The centimorgan distances for the .map files for each chromosome were interpolated using the Beagle utility program base2genetic.jar [34]. The output of the IBD calculations is a series of potential IBD segments shared between a pair of individuals containing the information of the physical base coordinates of first and last marker of the segment and the length of the segment in centimorgans. We used all segments > 2 cM to calculate the genome wide average and perform the permutation analysis to compare the IBD rates between pairs.

To detect any imbalance in the IBD rates, we calculated IBD proportion in case-case pairs as the fraction of pairs, computed from all case-case pairs, that are estimated to be identical by descent at a given location in the genome. Similarly, the IBD proportion in case-controls pairs was computed from all case–control pairs. The difference in IBD is the IBD proportion in cases minus IBD proportion in controls.

The IBD test evaluates the difference in IBD proportions between case-case and case–control pairs compared against a null distribution made from five million permutations of case and control status. Scripts used to perform the IBD test were previously published and kindly provided by the authors upon request [23]. Because of the limited number of permutations, the smallest *P* value detectable is 2 × 10^–7^. We calculated the difference in IBD proportions and the corresponding permutation *P*-value at every 10th SNP along the autosomes as described before [23]. In addition, we calculated permutation *P*-values genome-wide for 1000 permutations of case–control status, which allows us to determine the correct multiple-testing adjustment. The threshold for genome-wide significance was estimated as the 0.05 percentile of the distribution for the permutation *P*-values corresponding to *P* value = 1.30 × 10^–5^ and 1.26 × 10^–7^ for the individuals of European ancestry and admixed African Americans, respectively. These values are very similar to previously established genome-wide significance threshold for IBD mapping using a population of European ancestry individuals [16]. When calculating the *P* values in the permutation analysis, it was corrected for the average genome-wide sharing as recommended by the authors [16, 23]. We used customized R scripts to graph the distribution of the length of the detected IBD segments, IBD association results and to examine the distribution of IBD *P* values among the 22 chromosomes.

### Admixture mapping

To complement the IBD results, we investigated if at any given locus in the genome, there was a significantly different proportion of alleles of African vs. European ancestry between cases and controls in the admixed African American group. To estimate local ancestry, we assumed a two-ancestry model using 98 unrelated European (CEU) and 97 African (YRI) samples from 1000 Genomes phase 3 [38] as reference panels for phasing. We merged reference and HCV African American datasets using PLINK version 2.0 [14, 39] keeping 655,628 autosomal markers with overall genotyping rate of 0.99. Genetic and physical distances for this analysis were based on build GRCh37/hg19 of the human genome [36]. We used an LD based genetic map as described by Brown et al. [35] to be consistent with the original algorithm while being aware that LD-based maps as opposed to pedigree maps can create biases in regions where there is strong selection [40]. The original map was generated using LDhat (https://github.com/auton1/LDhat/) as described in reference [37]. We phased reference and HCV African American samples jointly using Beagle version 5.4 [34] and estimated local ancestry calls using RFMIX version 2 [41]. Proportions of African global ancestries were estimated by averaging the local ancestry estimates across all chromosomes. We calculated principal components and the genetic relatedness matrix (GRM) using the PC-AiR [42] and PC-Relate [43] methods implemented in the GENESIS R package [44]. PC-AiR uses measures of ancestry divergence estimated using KING-Robust algorithm [45] to partition samples into related and unrelated ancestry representative sets. Estimation of pairwise kinship coefficients adjusted for population structure were obtained using PC-Relate [43]. We obtained PCs robust to relatedness and ancestry adjusted GRM by estimating PCs starting with the GRM obtained in the first round of analysis. To test for association of African local ancestry and HCV clearance, we used an admixture mapping logistic mixed model implemented in LLAMA [46] as part of the GENESIS R package [44]. We built the null model of association between ancestry and HCV clearance incorporating the three main principal components and human immunodeficiency virus infection status as fixed effect covariates and the GRM as random effect and applied a score test for each local ancestry locus to evaluate its association with the trait. The genome wide significance threshold for this analysis (*P* value < 1.80 × 10^–5^) was obtained using an analytical approach implemented in the package STEAM [47] in R.

## Supplementary Information

Supplementary Material 1.

## Data Availability

Genotype data is available upon request at dbGaP with accession number phs000454.v1.p1. Python programs implementing the IBD test that we used for the HCV clearance data can be downloaded from http://faculty.washington.edu/sguy/ibdmapping.html.

## Abbreviations

IBD: Identical by descent
GWAS: Genome wide association study
HCV: Hepatitis C virus
PCA: Principal component analysis
GRM: Genetic relatedness matrix
SD: Standard deviation
