# Supplementary material for: Identity by descent and local ancestry mapping of HCV spontaneous clearance in populations of diverse ancestries
Source: BMC Genomics. 2025 Jul 12;26:661. doi: 10.1186/s12864-024-11076-6 (PMC12255019; doi:10.1186/s12864-024-11076-6)
Supplement: Supplementary file 1 — Supplementary Material 1. [file 12864_2024_11076_MOESM1_ESM.docx]

**Identity by descent mapping of HCV spontaneous clearance in populations of diverse ancestry**

**Supplementary Figures**

Supplementary Figure S1. Principal component analysis of the individuals with HCV clearance and persistence participants in the Extended HCV Genetic Consortium by genetically determined ancestry groups. From left to right is shown the analysis of African Americans and individuals of European Ancestry. X and Y axes represent the first two principal components (PC1 and PC2) as determined from the ancestry-specific principal component analysis. Individuals are categorized as HCV clearance (pink) and HCV persistence (blue).


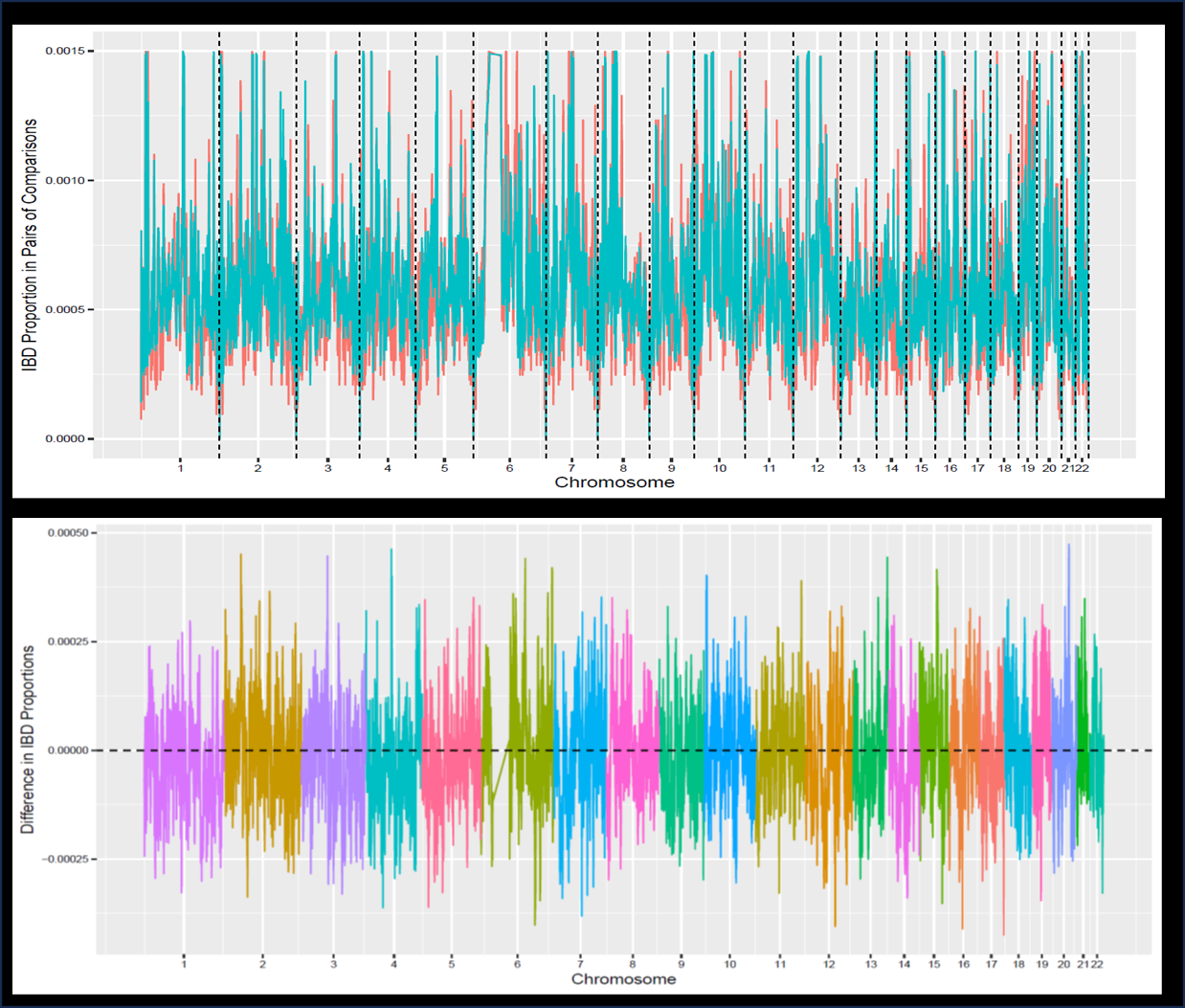


Supplementary Figure S2. The upper panel displays the IBD proportion in case-case pairs (pink) and case-control (green) across the genome in the African Americans. Values correspond to the fraction of pairs, computed from all case-case pairs and case-control pairs, that are estimated to be identical by descent at a given location in the genome. Dashed vertical lines mark chromosome boundaries (chromosomes 1-22). We observed a higher number of pairs in the boundaries of the chromosomes and centromere regions. The lower panel shows the difference between IBD proportion in case-case pairs minus IBD proportion in case-control pairs. Each chromosome is represented in a different color. The horizontal line indicates no difference (i.e., IBD proportion in cases= IBD proportion in controls), for comparison.

**
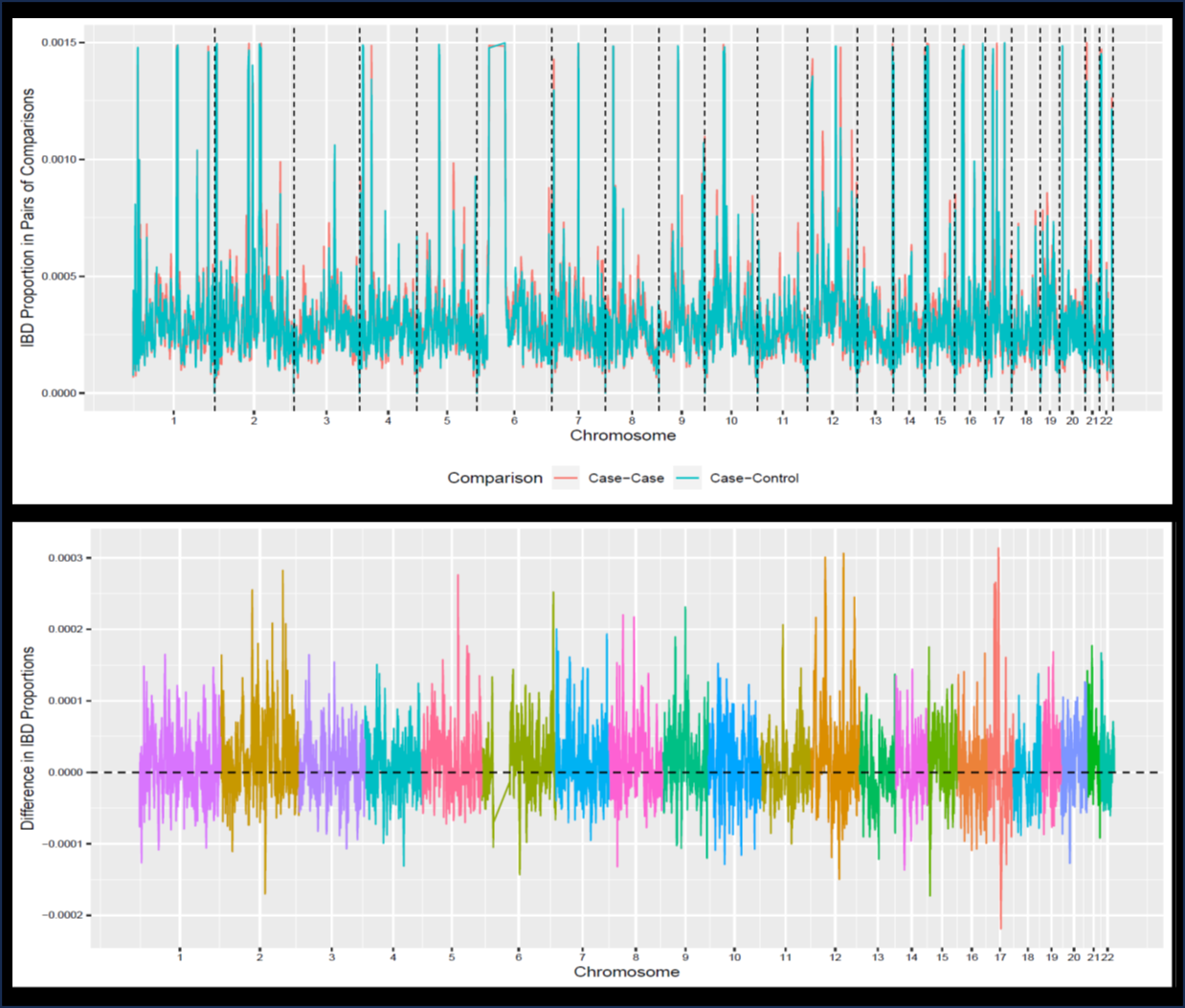
**

Supplementary Figure S3. The upper panel displays the IBD proportion in case-case pairs (pink) and case-control (green) across the genome in the individuals of European Ancestry. Values correspond to the fraction of pairs, computed from all case-case pairs and case-control pairs, that are estimated to be identical by descent at a given location in the genome. Dashed vertical lines mark chromosome boundaries (chromosomes 1-22). We observed a higher number of pairs in the boundaries of the chromosomes and centromere regions. The lower panel shows the difference between IBD proportion in case-case pairs minus IBD proportion in case-control pairs. Each chromosome is represented in a different color. The horizontal line indicates no difference (i.e., IBD proportion in cases= IBD proportion in controls), for comparison.
